# Effects of Long-Term Vegetation Restoration on Green Water Utilization Heterogeneity in the Loess Plateau Based on Field Experiments and Modeling

**DOI:** 10.3390/plants14050644

**Published:** 2025-02-20

**Authors:** Long Wang, Xiaoyu Song, Yu Liu, Lanjun Li, Xinkai Zhao, Pengfei Meng, Chong Fu, Wanyin Wei, Xuwu Wang, Huaiyou Li

**Affiliations:** 1 State Key Laboratory Base of Eco-Hydraulic Engineering in Arid Area, Xi’an University of Technology, Xi’an 710048, China; wl651552972@163.com (L.W.); li_lanjun@126.com (L.L.); xinkaizhao@126.com (X.Z.); mengpengfei2826@126.com (P.M.); 1210413023@stu.xaut.edu.cn (C.F.); weiwy7110@163.com (W.W.); 2220420111@stu.xaut.edu.cn (X.W.); 2 Water Conservancy Project Quality Safety Work Center of Yulin Water Conservancy Bureau, Yulin 719051, China; yuyu_xaut@163.com; 3 Xifeng Experiment Station of Soil and Water Conservation, Yellow River Conservancy Committee, Qingyang 745000, China; qyxflhy@sohu.com

**Keywords:** green water components, headwater catchment, forest and grass, rain-fed agricultural area

## Abstract

Due to the differences in the green water (GW) budget patterns of different vegetation, improper vegetation restoration may not only fail to improve the ecological environment but also cause irreversible damage to ecologically vulnerable areas, especially when vegetation restoration continues to be implemented in the future, and the pressure on water scarcity increases further. However, there is a lack of standardized research on the differences in the patterns of recharge, consumption, and efficient use of GW in typical vegetation. This makes the research results vary and cannot provide direct support for water management decision-making. Therefore, in this study, 30-year-old woodlands (*R. pseudoacacia* and *P. orientalis*) and two typical grasslands (*I. cylindrican* and *M. sativa*) that are similar to each other except for species were selected in a headwater catchment in the rain-fed agricultural area. A new GW concept and assessment framework was constructed to study the GW of long-term revegetation using a combination of field experiments and model simulations during the 2019–2020 growing season. The study findings comprise the following: (1) High-efficiency green water (GW_H_), low-efficiency green water (GW_L_), ineffective green water (GW_I_), and available green water storage (GW_A_) in the four sample plots during the study period were defined, separated, and compared. (2) An analysis of GW_A_ variations under different water scenarios. (3) The establishment of GW_H_ and GW_L_ thresholds. (4) Strategies to reduce GW_I_ and optimize GW potential while maintaining soil erosion prevention measures. (5) Suggestions for vegetation restoration species based on diverse factors. This research enhances comprehension of the impact of vegetation restoration on green water dynamics in ecologically vulnerable areas such as the rain-fed agricultural zone of the Loess Plateau.

## 1. Introduction

Green water (GW), which originates from precipitation, is stored in soil and subsequently enters the atmosphere through evaporation and transpiration, serves as a crucial link between land, terrestrial ecosystems, and the atmosphere [1]. In arid and semi-arid rainfed agricultural areas, GW plays a crucial role in supporting and regulating natural cycling processes within the terrestrial biosphere (such as energy and matter flows) and facilitating human activities (such as food production and ecological conservation), in contrast to blue water (which includes surface water and groundwater in the saturated zone) [2,3,4]. Studies have demonstrated that the cycling process of GW is influenced by a multitude of factors related to land use and climate change, encompassing topography, soil properties, precipitation patterns, temperature variations, and vegetation coverage [5,6]. Among the factors related to land use, vegetation coverage exerts the most significant influence on GW. The processes by which different vegetation types influence GW involve the interface between the atmosphere and the surface soil, leading to pronounced eco-hydrological effects [7]. Under different vegetation restoration modes, rainfall loss, interception, evapotranspiration, infiltration, and runoff are influenced by morphological characteristics [8]. Because of the vulnerability of GW to human activities represented by anthropogenic land-use change [9], perturbations to GW will realize off-site effects through the intra-terrestrial cycle. They will have a significant impact on precipitation (*P*), runoff, water resources, and even human activities in the Downwind Region [10]. Studies have shown that a complete understanding of the distribution characteristics and transformation mechanisms of GW in the ecologically vulnerable rain-fed agricultural areas located in headwater catchments of the Loess Plateau (LP) is crucial for vegetation restoration, regional ecological protection, and socio-economic development [11].

Researchers have conducted extensive studies focusing on various aspects, including the GW footprint of different crops and vegetation, the temporal and spatial variations in distribution, as well as the response of GW to changing environmental conditions [12,13,14,15,16,17]. Previous studies, however, have been largely independent, focusing primarily on water use patterns and their biophysical regulatory processes of vegetation in relation to soil moisture variations across regions. Their methodologies and conclusions vary widely, potentially hindering decision-making processes. To obtain valid information, it is essential to adhere to controlled variables (e.g., similar planting density, tree age, inflow, and soil properties). Consequently, pot experiments are often employed for studying multiple typical vegetation GW in the same region, lacking field-based research with uniform standards. Moreover, possibly due to the convenience of watershed-scale studies, existing research on green water components (GWCs) is somewhat simplistic. In these studies, GW is typically categorized into GW flow (evapotranspiration) and GW storage (soil moisture) and managed through soil moisture conservation, vapor transfer optimization, and water-saving measures [4,18]. However, GW flow can be further divided into evaporation and transpiration, which evidently differ in their support efficiency for vegetation. Some scholars have recognized this issue [19,20], further segregating GW flow into high-efficiency green water (GW_H_) and low-efficiency green water (GW_L_). Nevertheless, they have overlooked the interception of vegetation, neglecting its potential for GW conversion. Regarding GW storage, a pivotal oversight lies in the disregard for the fact that the portion of water residing below the wilting point is inaccessible for plant absorption and utilization, thereby resulting in an inflated assessment of GW availability. Consequently, to attain accurate management of GW resources, it is imperative that a novel methodology for categorizing GWCs be introduced.

In the rain-fed agriculture area of the LP, there are two main vegetation restoration types—artificial woodland and grassland. *R. pseudoacacia* and *P. orientalis* are the most common species in the artificial woodland, while *I. cylindrica* and *M. sativa* dominate the natural restoration and artificial grassland, respectively. There is disagreement among scholars over the most suitable species for future vegetation restoration on the LP, as different vegetation types perform differently in soil and water conservation, ecological environment improvement, and water resource utilization [21,22,23,24]. The “Grain for Green” project, which is an essential measure of environmental management in the LP region, will continue to be promoted in the next 30 years. However, the pressure on water resources in the region may further intensify [25]. Comparative studies on the ecological functions of different vegetation from the fundamental perspective of GWCs and their comprehensive performance are crucial for the future direction of vegetation restoration in the typical rain-fed agriculture area of the LP. As the headwater catchment serves as the starting point for the convergence and generation of water and sediments, it is critical for downstream water resource utilization and ecological protection that research on the above issues be carried out in this area first. In addition, even if suitable vegetation restoration types are identified, policymakers may be unable to formulate practical and feasible targeted policies or implementation programs in the face of multi-objective vegetation restoration requirements due to the lack of detailed and reliable data from multi-perspective studies. Therefore, in response to the above problems, researchers should also consider how to implement effective vegetation management and develop easy-to-use implementation programs to ensure efficient use of water resources and sustainable development of ecosystems.

In summary, the Nanxiaohegou Basin, a typical source watershed of the rain-fed agriculture area of the LP, which has been managed since 1951, was selected for this study. Typical long-term vegetation restoration sample plots (>30 years) with similar planting densities, age, and slope orientation were selected in this watershed, and high-resolution precipitation, interception, soil evaporation, and soil moisture content data were obtained through continuous field trials and fixed-point observations during the two plant growing seasons of 2019–2020 (15 April to 15 October of each year). Combined with crop growth and water demand model simulations, a new framework for classifying and assessing GWCs of restoration vegetation based on field experiments was constructed to distinguish the performance of different vegetation in terms of water retention capacity, GW use efficiency, and potential enhancement. The aim is to reveal the GW transformation mechanism of different vegetation on the LP and the differences in their hydrological and ecological functions, and analyze the prospect of GW resource development and utilization.

## 2. Description of Sample Plots and Methods

### 2.1. Study Site and Sample Plots Selection

**Figure 1:**
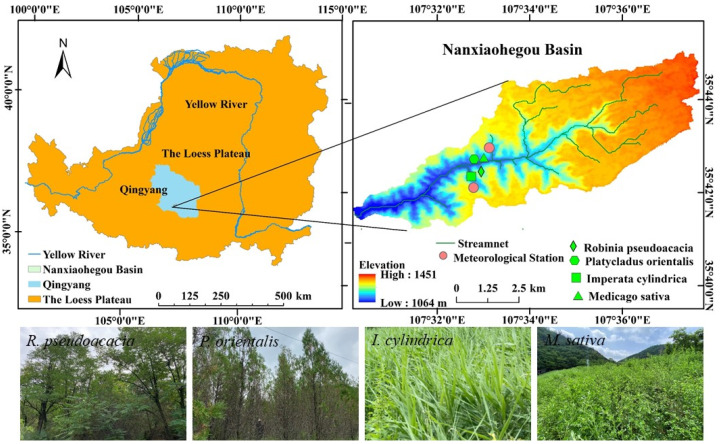
Location of the NXHG Basin, meteorological station, and sample plots.

The Nanxiaohegou Basin (NXHG Basin) (107°30′–107°37′ E and 35°41′–35°44′ N, 36.2 km^2^), a typical semi-arid rain-fed area, was selected for this study. The basin is located in the central part of the Loess Plateau (Figure 1). It is a soil and water conservation pilot and demonstration area selected by the Yellow River Conservancy Committee. The region has a typical warm-temperate continental climate, with an average annual precipitation of 546.7 mm (1970–2020), of which more than 84.9% is due to short rainstorms during the growing season (15 April to 15 October each year). After more than 72 years of management practices, the vegetation cover increased from 1.3% in 1954 to 39.28% in 2020, with the main tree species including *Robinia pseudoacacia* (*R. pseudoacacia)*, *Platycladus orientalis* (*P. orientalis*), and a small number of economic forests such as peach and jujube. The main grasses include *Imperata cylindrica* (*I. cylindrica*, natural grassland) and *Medicago sativa* (*M. sativa*, artificial grassland). The basin is geologically homogeneous, and the dominant soil type is loess, which can reach depths of 50 to 200 m. Influenced by long-term soil erosion, the main landforms in the basin include loess, rounded slopes, and gullies.

### 2.2. Field Experiment Design

#### 2.2.1. Sample Plot Measurement

We surveyed the selected sample plots in April 2019 to obtain the data for this paper. Stand parameters were determined within each sample plot using a slope meter (Digipas Technoligies Inc., ACE-SP400C, Shanghai, China) and a high-precision hand-held GPS (Kemai Instruments Co., Ltd., KM-6A, Shenzhen, China). In order to accurately represent the *R. pseudoacacia* and *P. orientalis* sample plots, 10 trees were randomly selected as samples. The following parameters were measured for each representative plant: diameter at breast height (DBH), tree height, canopy amplitude, and coverage. The average values of these parameters for the 10 representative plants were used as the values for the entire sample plot. The specific data can be found in Table 1.

The standing conditions of *I. cylindrica* and *M. sativa* were measured using the same method as for the woodlands. Ten points were selected for measuring vegetation parameters in each of the two grasslands. Monthly measurements of plant height and cover were taken in the two sample plots from 15 April to 15 October during the growing seasons of 2019 and 2020. The representative value for each measurement point was determined by calculating the average, and the specific statistical data are presented in Table 2.

#### 2.2.2. Fixed-Point Observation

The study employed automatic fixed-point monitoring, manual fixed-point monitoring, and indoor measurement to collect the required data. Two small-scale meteorological reporting systems (Watchdog series 2009, Shenzhen, China) were used to obtain meteorological data, such as rainfall, temperature, wind speed, and solar radiation, through automatic spot monitoring with an observation period of one hour. The data was exported twice a year, on 1 July and 16 October. The instruments were regularly checked to ensure the accuracy and continuity of the monitoring.

GW hydrologic processes within each sample plot were monitored using sentinel observations for the 2019 and 2020 growing seasons. These observations included canopy interception of tree vegetation, soil evaporation, soil water content, leaf area index (LAI), and vegetation root density distribution. We placed 1–2 rain barrels with a diameter of 20 cm and a depth of more than 20 cm under selected representative plants in each woodland plot. After each rainfall, we recorded the rainfall data and used the average value as the penetrating rainfall. The difference between this value and the actual rainfall was the interception. Soil evaporation was carried out using a manufactured miniature evapotranspiration meter with an internal diameter of 16 cm and a depth of 27 cm. Three such meters were set up for each sample plot, and the average value was also taken. Soil moisture content was measured using a tube time-domain reflectance system (Trime TDR, Germany) at two randomly selected locations, 50 cm away from the main trunk of the representative plant, with a depth of 0–200 cm. Measurements were taken at 10 cm intervals from 0 to 100 cm depth and at 20 cm intervals from 100 to 200 cm depth. The mean value of the two measurements was taken. Soil evaporation and moisture content were observed at intervals of 1 to 7 days, with an additional measurement taken on the day after rain. The plant canopy analysis system (WinScanopy 2006, Montreal, Richmond, Canada) was used to conduct fixed-point LAI observations during each plot’s growing season. Three fixed positions and angles were selected for each sample plot, and observation intervals were 5–10 days with the same values taken as above.

The initial values of soil physical parameters were measured at depths of 20 cm, 70 cm, and 160 cm, which were used to represent the average conditions of the 0–40 cm, 40–100 cm, and 100–200 cm soil layers, respectively. Soil samples were collected from a selected plant in each sample plot, considered the representative measurement point, at a distance of 50 cm from its trunk. These samples were then processed and taken to the laboratory for analysis. The composition of soil particles was measured using a laser particle size analyzer (Mastersizer 2000, Malvern, Worcestershire, UK). Based on this analysis, the residual moisture content was determined using the Rosetta model [19]. The study determined the saturated hydraulic conductivity of the soil in each layer using the constant head method [19]. The soil moisture characteristic curve was determined by centrifugation. In addition, the soil bulk weight, saturated water content, field water capacity, and capillary fracture water content were determined using the cutting ring. The soil auger method was used to select three sampling points at the same location for soil sampling. Finally, root distribution density was determined indoors for each plot.

### 2.3. Hydrus-1D Model

#### 2.3.1. Model Description

The HYDRUS model (version 5.06) is software that was developed by the National Salt and Soil Laboratory of the U.S. Department of Agriculture (USDA) to simulate water movement, heat transport, and solute transport in both saturated and unsaturated media. It is widely used for soil moisture dynamics simulations, as well as solute transport and vegetation evapotranspiration simulations [20]. In this study, rainfall allocation simulation under vegetation conditions was carried out on terraces. Since the water movement is mainly concentrated in the vertical direction, we chose the Hydrus-1D model for simulation. This model is simple to operate, easy to obtain, and free of charge. It describes soil water movement using the modified Richards equation embedded with a source-sink term and simulates the root water uptake process using the Feddes model. For more information on the equations and models for soil evaporation, vegetation transpiration, and surface runoff simulations, please refer to [26].

#### 2.3.2. Model Boundary and Initial Condition Settings

The area for simulation in the model is the soil layer of 0–200 cm, where plant roots absorb water. The simulation step is set as 1 day, and the initial condition is based on the soil moisture measured on 14 April every year. To account for the local groundwater depth and the actual water cycle, the model’s upper and lower boundaries were set as the atmospheric boundary with runoff and the drainage boundary, respectively.

#### 2.3.3. Model Calibration and Validation

In the study conducted by [19] to simulate evapotranspiration in the NXHG Basin, a sensitivity analysis of Hydrus-1D model parameters was performed. In this paper, the simulation performance was evaluated using the Nash–Sutcliffe Efficiency Coefficient (*NSE*), with the observed soil moisture content and soil evaporation data measured during the test period. The year 2019 was used as the calibration period, and 2020 was used as the validation period.

The calibrated model was used to simulate the process of water transport and transformation in the four sample plots in 2020. The model was also used to calculate the allocation of rainfall by vegetation and to validate the model based on the observed data. It should be noted that in areas with loess soil, groundwater is buried deeper and not involved in the soil water cycle. Therefore, its role in recharging the surface and shallow soil moisture through the rising action of the capillary can be ignored. The recharge, storage, and consumption of GW mainly occur during rainfall, soil moisture variability, and evapotranspiration. When the model can effectively simulate soil moisture content and evaporation, it can also accurately simulate vegetation transpiration.

#### 2.3.4. Green Water Components Separation

**Figure 2:**
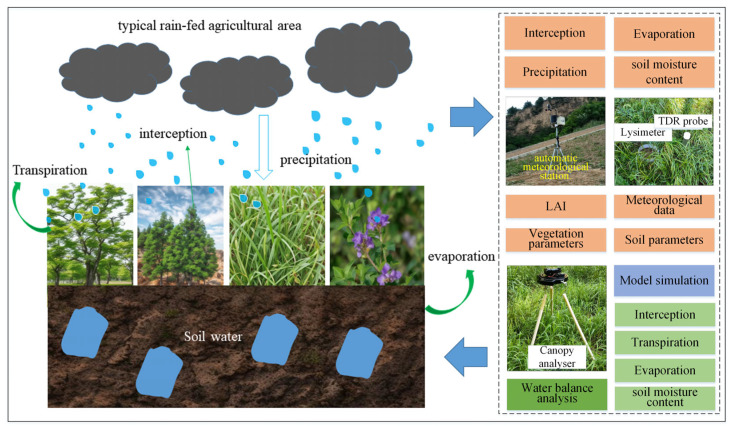
Framework chart for the green water components study.

The authors previously proposed a new basin-scale green water components concept that further classifies GW into high-efficiency green water (GW_H_), low-efficiency green water (GW_L_), ineffective green water (GW_I_), and available green water storage (GW_A_) [27]. The introduction of this concept has refined GW research and led to an increase in the accuracy of GW assessment. In this paper, it is improved and used as a core indicator in the green water assessment framework for long-term vegetation restoration samples. The flow chart for the green water components study is shown in Figure 2. 

(1) High-efficiency green water (GW_H_): Actual vegetation transpiration was calculated using Equations (1)–(6).

(1) $$T_{P}=ET_{0}e^{-\mu LAI}$$

where *T_p_* is potential transpiration, *ET*_0_ is potential evapotranspiration, *μ* is the crop extinction coefficient, and *LAI* is the leaf area index.

(2) $$ET_{0}=\frac{0.408\Delta (Rn-G)+\gamma \frac{900}{T+273}U_{2}(e_{a}-e_{d})}{\Delta +\gamma (1+0.34U_{2})}$$

where ∆ is the slope on the saturated water vapor pressure–temperature curve (kPa-K^−1^). *R_n_* is the net solar radiation (J·m^−2^·d^−1^), and *G* is the soil heat flux (J·m^−2^·d^−1^). *e_a_* is the saturated water vapor pressure (kPa). *e_d_* is the actual water vapor pressure (kPa), *γ* is the hygrometer constant (kPa·K^−1^), and *U*_2_ is the wind speed at a distance of 2 m from the surface (m/s).

(3) $$\beta \left(z\right)=\frac{\beta^{\prime }\left(z\right)}{\int_{0}^{Lr}\beta^{\prime }\left(z\right)dz}$$

where *β*(*z*) is the measured root distribution function, and *Lr* is the root distribution depth.

(4) $$\alpha (h)=\left\{\begin{array}{ll} h/h_{1} & h_{1}\leq h\leq 0\\ 1 & h_{2}\leq h\leq h_{1}\\ \left(h-h_{3}\right)/(h_{2}-h_{3}) & h_{3}\leq h\leq h_{2}\\ 0 & h<h_{3} \end{array}\right.$$

where *h*_1_, *h*_2,_ and *h*_3_ are the three water potential thresholds affecting the water uptake by the root system of the vegetation (cm). *h*_3_ is the soil water potential when permanent wilting of the crop occurs, *h*_2_ is the soil water potential corresponding to the beginning of the decrease in the rate of water uptake by the root system, and *h*_1_ is the soil water potential when the rate of water uptake by the root system decreases due to high soil moisture.

(5) $$S_{r}\left(z,t\right)=T_{P}\int_{0}^{Lr}\beta \left(z\right)\alpha \left(h\right)dz$$

where *S_r_*(*z*,*t*) is the actual rate of crop transpiration.

(6) $${\mathrm{GW}}_{H}=S_{r}(z,t)\cdot T$$

(2) Low-efficiency green water (GW_L_): Actual soil evaporation was calculated using Equations (7)–(9).

(7) $$E_{P}=ET_{0}(1-e^{-\mu LAI})$$

(8) $$E_{r}\left(0,t\right)=\left\{\begin{array}{cc} E_{p} & \theta \geq 0.65\theta_{f}\\ E_{p}\left(0.65\theta_{f}-\theta \right)/\left(0.65\theta_{f}-\theta_{C}\right) & \theta_{c}\leq \theta <0.65\theta_{f}\\ 0 & \theta <\theta_{c} \end{array}\right.$$

(9) $${\mathrm{GW}}_{L}=E_{r}\left(0,t\right)\cdot T$$

where *E_p_* is potential evaporation, *E_r_*(0,*t*) is the actual rate of soil evaporation, *θ* is the water content of the surface soil, *θ_f_* is the field moisture capacity, and *θ_c_* is the moisture content of capillary fracture.

(3) Ineffective green water (GW_I_): Interception. Rainfall consumption and evaporation intercepted by vegetation reduce the effective rainfall and soil moisture recharge reaching the surface, and represent an unproductive loss of green water resources. It was calculated using Equations (10) and (11).

(10) $${\mathrm{GW}}_{I}=aLAI\left(1-\frac{1}{1+\frac{bP}{aLAI}}\right)$$

(11) $$b=1-e^{-\mu LAI}$$

where *P* is rainfall, and *a* is an empirical parameter with an initial value of 0.25.

(4) Available green water storage (GW_A_): As water below the wilting water content is difficult for plants to absorb, the difference between the actual soil water content and the wilting water content is taken as the amount of unused green water resources and is calculated as follows.

(12) $${\mathrm{GW}}_{A}=\sum_{i=1}^{n}SWi-Ww,i$$

where *SW_i_* and *W_w,i_* are the number of GW_A_, the actual moisture content of the soil evaluation layer *i*, and the wilting water content of evaluation layer *i*, respectively.

Based on the above definition of GWCs, we can use the Hydrus-1D model to extract them and then analyze the methods and mechanisms by which vegetation allocates GW resources.

### 2.4. Data Processing and Analysis

Statistical calculations and analyses were carried out on the GWCs of various plots, which included GW_H_, GW_L_, GW_I_, and GW_A_. Regression methods, whether linear or nonlinear, were utilized to determine the relationships between GW_H_ and GW_L_ of multiple plots and their influencing factors, followed by comparative analysis and threshold determination. Multivariate regression analysis was applied to analyze how to reduce GW_I_ and enhance GW potential, with a significance level of 0.01. Unlike univariate linear regression, which considers only one predictor variable, multivariate linear regression uses several predictor variables simultaneously to predict the value of the dependent variable, thereby identifying the joint effects of these predictor variables. The analysis results reveal the explanatory power of different factors influencing GW_I_. Descriptive statistics and multivariate regression analysis were performed using SPSS 21.0 statistical software (IBM SPSS, Inc., Armonk, New York, USA). Fitting functions and plotting were completed using Origin 2019 (OriginLab Corp., Northampton, Massachusetts, USA).

## 3. Results

### 3.1. Model Calibration and Validation Results

#### 3.1.1. Results of Parameter Sensitivity Analysis and Calibration

The results for each plot’s calibrated model parameters sensitivity ranking are presented in Table 3.

Based on the sensitivity ranking results, it is evident that the parameter of pore size distribution has the greatest influence on GW simulation across all four vegetation sample plots. The second most sensitive parameter is the saturated moisture content. Additionally, the sensitivity of each parameter varies for different types of vegetation.

#### 3.1.2. Model Validation Results

The calibrated model was used to simulate the water transport and transformation process within the four sample plots in 2020, calculate the allocation of rainfall by vegetation, and validate the model based on the observed data.

**Figure 3:**
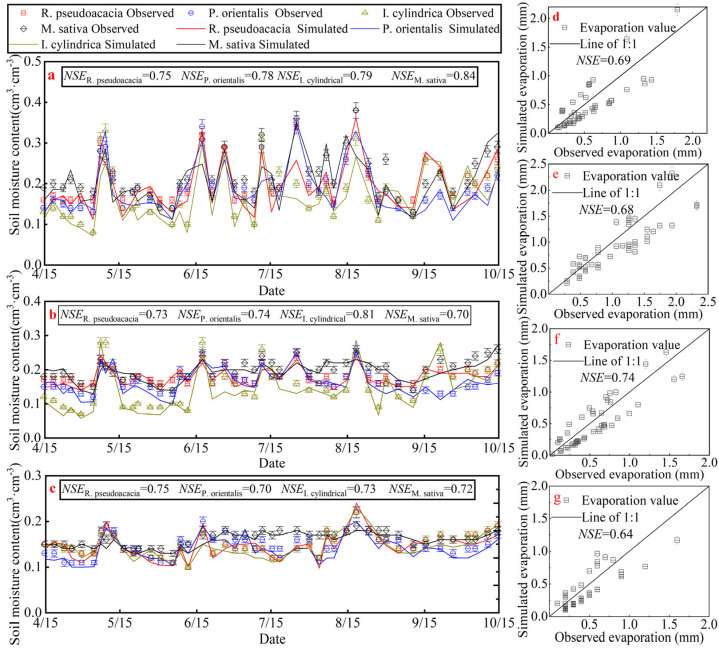
Model validation results for soil moisture content and soil evaporation in four plots. (**a**–**c**) are measured, simulated, and *NSE* of soil water content at 20, 70, and 160 cm depths of the four sample plots, respectively; (**d**–**g**) are measured, simulated, and *NSE* of soil evapotranspiration for *R. pseudoacacia*, *P. orientalis*, *I. cylindrica,* and *M. sativa*, respectively.

According to the comparison between the simulation results, measured data, and *NSE* in Figure 3, it can be seen that in the simulation of soil moisture at a depth of 20 cm, except for *R. pseudoacacia*, the *NSE* of the other three plots is greater than 0.75, and the simulation effect is “very good”. In the simulation of soil moisture at a depth of 70 cm, except for the *NSE* of *I. cylindrica*, the *NSE* of the other three sample plots is less than 0.75 and greater than 0.65, and the simulation effect is “good”. At 160 cm, the *NSE* of the four plots were all less than or equal to 0.75 and greater than 0.65, and the simulation effect was “good”. For the soil evaporation simulation, the four plots’ performance was “good”. This indicates that the Hydrus-1D model can well realize the simulation of the GW migration and transformation process.

### 3.2. Separation Results of Green Water Components

**Figure 4:**
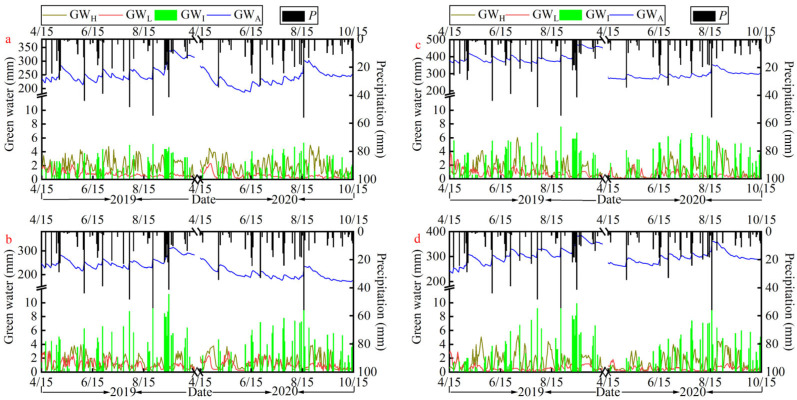
Number of GWCs and precipitation (*P*) during the growing season in the four sample plots. (**a**–**d**) represent the number of green water components for the 2019–2020 growing season for the four vegetation plots of *R. pseudoacacia*, *P. orientalis*, *I. cylindrica*, and *M. sativa*, respectively.

After calibrating and validating the Hydrus-1D model, it is possible to simulate and output the daily scale GW hydrological cycle process in each sample plot. Based on the model output data, the dynamic characteristics of the number of each GWC in the growing season can be analyzed. By using the above methods, it is also possible to overcome the defects of discontinuity in the observation of each GWC, which leads to errors in the assessment of GW. The results of the number of GWCs in each plot during the growing season are shown in Figure 4.

The main precipitation for the 2019–2020 growing season is concentrated in July–September, with annual rainfall of 658.4 and 480.0 mm in 2019 and 2020, respectively. According to Figure 4, the GWCs show a fluctuating trend in all four vegetation sample plots. From the distribution of GWCs in the growing season of each plot, the amount of GW_H_ was lower on rainy days, while it was enough to maintain a higher level on rain-free days following precipitation. In the early growth stage (15 April to 15 May), GW_H_ was generally smaller than GW_L_, while in the middle (16 May to 31 August) and late growth stages (1 September to 15 October), GW_H_ was significantly larger than GW_L_, which varied substantially with precipitation, while GW_L_ remained at a lower level and stable values. The amount of GW_I_ was closely related to the amount of rainfall, and in the early and late stages of the growing season, there was less rainfall, and the value of GW_I_ was relatively low. However, the average value in the late growth stage was significantly higher than that of the early, which may be related to the LAI and canopy density. Moreover, among the four plots, the GW_I_ of *R. pseudoacacia* was not only significantly lower than that of *P. orientalis* but also lower than that of the two grassland plots under the same rainfall. GW_A_ of *R. pseudoacacia* was significantly higher in the early and late growth stages than in the middle, while the other samples showed small fluctuating changes. From the relationship of GW_A_ with GW_H_ and GW_L_, it can be seen that in the mid-growth stage, the expenditure of GW_A_ was mainly controlled by GW_H_.

For the *R. pseudoacacia* plot (Figure 4a), the day-by-day GW_H_ was distributed at 0.4–4.8 and 0.2–5.0 mm, GW_L_ at 0.2–2.1 and 0.1–2.3 mm, GW_I_ at 0.1–5.1 and 0.1–5.3 mm, and GW_A_ at 214.1–343.7 and 186.6–302.4 mm over the two experimental growth seasons. The *P. orientalis* (Figure 4b) had four GWC counts, distributed at 0.1–3.9 and 0.3–3.9 mm, 0.2–2.9 and 0.2–3.0 mm, 0.1–11.3 and 0.1–11.1 mm, and 215.2–313.8 and 170.3–269.0 mm, respectively, over the two growing seasons. Compared to *R. pseudoacacia*, *P. orientalis* had lower values of GW_H_ and GW_A_ in both growing seasons, while GW_L_ and GW_I_ were higher. When looking at the amount of GWCs in both stands during both growing seasons, it was found that the amount was greater in 2019 than in 2020. This difference may be due to the fact that 2019 received 37.2% more precipitation than 2020.

For grasses, *I. cylindrica* (Figure 4c) had day-by-day GW_H_ distributions of 0.3–6.0 and 0.0–5.3 mm, GW_L_ distributions of 0.1–3.8 and 0.1–2.2 mm, GW_I_ distributions of 0.1–7.6 and 0.1–6.7 mm, and GW_A_ distributions of 360.3–474.2 and 267.2–352.8 mm over the two experimental growing seasons. *M. sativa* (Figure 4d) had four GWCs quantities distributed at 0.1–5.3 and 0.2–4.5 mm, 0.1–3.0 and 0.1–1.8 mm, 0.1–10.3 and 0.1–9.9 mm, and 234.4–387.0 and 260.0–361.0 mm, respectively, over two growing seasons. In terms of the mean values, the GW_L_ and GW_A_ of *I. cylindrica* in the 2019 growing season were greater than that of *M. sativa*, and the GW_H_ and GW_I_ were slightly lower than that of *M. sativa*. In the 2020 growing season, the GW_L_ of *I. cylindrica* was still greater than that of *M. sativa,* while GW_A_, GW_H_, and GW_I_ were lower than that of *M. sativa*. Comparing the quantities of each GWC in both *I. cylindrica* and *M. sativa* plots separately, it can be found that the quantities of all three GWCs except GW_I_ were higher in 2019 than in the 2020 growing season, especially in the GW_A_ of *I. cylindrica*.

From the data of various GWCs of the four plots, it can be seen that the recharge of GW_A_ comes from precipitation (the loess is deep enough for groundwater recharge to be neglected), while the expenditure is controlled by different processes at different stages. Among the four plots, *P. orientalis* had the smallest average value of GW_A_, the highest interception rate, and too much GW_I_, which amounted to 30.1% and 36.6% of precipitation. *R. pseudoacacia* had the most GW_H_ and the least GW_I_ of the four plots. *I. cylindrica* had the most GW_A_, 28.7% higher than *M. sativa*, which is also grass, and 56.8% higher than *P. orientalis* during the 2019 growing season when precipitation was higher, and may have the greatest ability to hold water of the four vegetation species. *M. sativa* had less GW_I_ than *P. orientalis* and spent more on GW_H_ than *I. cylindrica* during the experimental period.

### 3.3. Green Water Allocation Patterns for Different Vegetation

**Figure 5:**
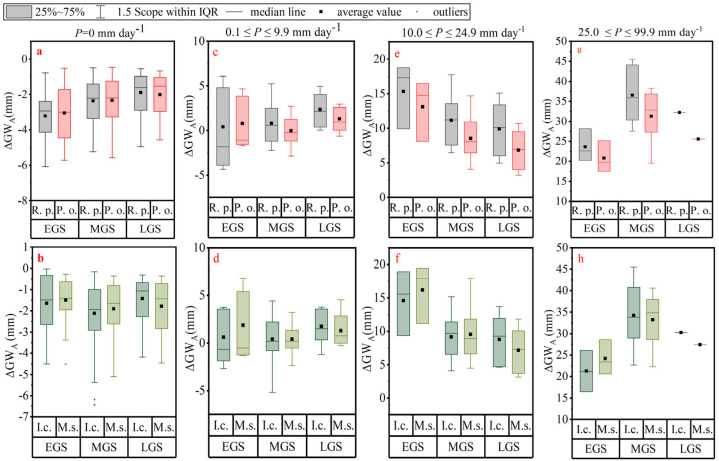
Variation in available green water storage for each sample plot. (**a**–**h**) show the change in GW_A_ for woodland sample plots (*R. pseudoacacia* and *P. orientalis*) and grassland sample plots (*I. cylindrica* and *M. sativa*) for rain-free days, light rain, moderate rain, and heavy rain or more in the early, middle, and late growing seasons, respectively. Box plots show the median, quartiles, and possible outliers of the data. The boundaries of the box are the 25th and 75th percentiles, and the box whisker extends to a range within 1.5 IQR (Interquartile Range). Data points that extend beyond this range are individually marked on the box plot as potential outliers. Note change in scale for each rainfall amount.

GW variability is closely related to growth stage and rainfall. Therefore, the growth season is divided into three stages: early growth (15 April to 15 May), middle growth (16 May to 31 August), and late growth (1 September to 15 October). Rainfall will be divided into four levels according to size: rain-free days, light rain, moderate rain, and heavy rain or more, to analyze changes in GW_A_. The results are shown in Figure 5.

GW_A_ varied with growing season and rainfall at each plot (Figure 5). On rain-free days, the changes in GW_A_ were negative, and the absolute value of the mean of *R. pseudoacacia* was greater than that of *P. orientalis* in the early and middle growth stages. The range of the changes in GW_A_ of *P. orientalis* was greater than that of *R. pseudoacacia* in the early growth stage, and then they were close to each other. GW_A_ changes for the grasses were also all negative, with the absolute value of the mean of the changes being greater for *I. cylindrica* than for *M. sativa* in the early and middle growth stages and less in the late stage. In terms of the range of changes, *I. cylindrica* was progressively decreasing, while *M. sativa* was increasing.

When the rainfall was light, the change in GW_A_ in the woodland had both positive and negative values. The change in GW_A_ in both *R. pseudoacacia* and *P. orientalis* was close to zero in the early and middle growth stages; however, the range of GW_A_ in the early growth stage was greater than that in the middle. *I. cylindrica* and *M. sativa* also showed similar characteristics, except that in the early growth period, *M. sativa* was significantly more recharged with GW_A_ than *I. cylindrica* at the same time due to smaller plant size, lower transpiration and interception rates, and comparable soil evaporation. Interestingly, with the exception of *M. sativa*, GW_A_ recharge was slightly greater in the late growth stage in other plots than in other stages. After analyzing the rainfall, GW_H_, and GW_L_ data, it was observed that even though the LAI and canopy density were at their highest, the GW_A_ increased due to the reduced GW expenditures caused by lower temperatures during the late period and higher and more concentrated rainfall in 2019.

GW_A_ was replenished after each moderate or heavy rain event. The average increase in GW_A_ for both woodland and grassland at moderate rainfall levels was early > middle > late growth stage, with *R. pseudoacacia* recharging more GW_A_ than *P. orientalis* in the woodland. This is because *P. orientalis* has more GW_I_ than *R. pseudoacacia*, which directly reduces the amount of rain reaching the ground. During the grassland growth stages, the GW_A_ recharge of *M. sativa* was greater than *I. cylindrica*, except in the late growth stage. Even when the rainfall was ≥25 mm, the soil moisture content of *R. pseudoacacia* was still higher than that of *P. orientalis* throughout the entire growth season. In the middle and late growth stages, the GW_A_ recharge of *M. sativa* was smaller than that of *I. cylindrica*.

## 4. Discussion

### 4.1. Effects of Different Vegetation Types on Water Conservation Function

The “Grain for Green project” has been proven to be an effective way to improve the ecological environment in the LP. It has become the consensus of many scholars that the increase in forest and grass cover effectively reduces soil erosion and curbs land degradation, especially in the gully areas of the LP [28,29]. However, forests and grasses also influence the replenishment (rainfall reaching the ground and the physicochemical properties of the soil) and consumption (evapotranspiration process) of soil moisture by changing the process of rainfall distribution, and some scholars believe that forests and grasses play an important function of water conservation [20,30]. It has also been argued that the increasing vegetation cover is a “water pump”, leading to the emergence of a dried soil layer on the LP, which is at risk of intensification, especially in areas of long-term vegetation restoration [31]. Precipitation is the only source of water recharge in the typical rain-fed agricultural areas of the LP, and the above controversy should be emphasized even more in this region.

There is a tightly coupled relationship between GW_A_ and vegetation, with GW_A_ supporting the growth and development of vegetation, which in turn influences soil water distribution through its own form and function [32]. Furthermore, GW_A_ is influenced by a multitude of factors, including climate, vegetation type, tree age, planting density, and topographical conditions [33]. Based on the study results, we have found that GWCs exhibit varying behaviors across different growing seasons and vegetation samples. Furthermore, the direct source of evapotranspiration is the soil-stored GW. The variation of GW_A_ indicates that different vegetation has various GW allocation patterns (Figure 5), which also represents the difference in the performance of vegetation’s water conservation function [34]. The consumption of GW_A_ by GW_H_ and GW_L_ varies to a certain extent. Current studies on soil moisture changes in different vegetation mostly consider multi-year scales [30]. Due to the differences in the spatial and temporal distribution of precipitation and the difficulty of long-term high-precision monitoring of GW, related studies have neglected the effects of the stage of vegetation development and the amount of rainfall on soil moisture replenishment and expenditure [7]. However, from the results, we can find that the changes of GW_H_, GW_L_, GW_A_, and GW_I_ of the four typical long-term restoration vegetation in different growth periods and under different rainfall conditions also show different characteristics.

This study shows that on rain-free days, GW expenditures were slightly greater for *R. pseudoacacia* than for *P. orientalis* during the growing season, and greater for *I. cylindrical* than for *M. sativa*, except in the late growth stage. When rainfall was light, the water conservation capacity of *R. pseudoacacia* was greater than that of *P. orientalis*, and that of *M. sativa* was greater than that of *I. cylindrical* except in the late growth stage. During moderate rainfall, the water conservation capacity of *R. pseudoacacia* was greater than that of *P. orientalis* throughout the growth cycle, and the water conservation capacity of *M. sativa* was greater than that of *I. cylindrica* at all times, except in the late growth stage. When rainfall was heavy or more, *R. pseudoacacia* still led in the water conservation function, and *M. sativa’s* water conservation capacity was lower than that of *I. cylindrica*, except during the early growth stage.

In addition, research has shown that tree age is a major factor influencing GW_A_ under similar standards and climate [33]. For *R. pseudoacacia*, the variation in GW_A_ among saplings aged 5 to 10 years is significantly smaller compared to those of other age groups [35]. With the increase in planting duration, the degree of variation in GW_A_ across different soil layers in *R. pseudoacacia* and *P. orientalis* becomes significantly more pronounced. This is attributed to the more extensive development of their root systems as the vegetation matures. When water availability is abundant, *R. pseudoacacia* and *P. orientalis* preferentially utilize the soil moisture within the 0–100 cm layer. *R. pseudoacacia* aged 0–20 years have the most significant impact on soil moisture in the 0–100 cm layer, which aligns with the findings of this study. In response to variations in rainfall, *R. pseudoacacia* and *P. orientalis* exhibit high sensitivity and are able to promptly adjust their water absorption depths. The changes in GW_A_ observed in this study further corroborate this point. Reference [35] pointed out that when the tree age exceeds 20 years, with canopy density remaining unchanged, the overall growth and health status of *R. pseudoacacia* continues to decline due to water stress, implying a deterioration of its ecological functions over time. According to the results (Figure 3c), when water demand increases or water availability decreases, the response degree of GW_A_ within the 100–200 cm soil layer intensifies. Reference [36] discovered that the daily average transpiration rate of 30-year-old *R. pseudoacacia* in their study was lower than that of other broadleaf species, manifesting as canopy wilting and slow growth. The primary cause of this phenomenon was severe soil water stress. However, based on the soil water content threshold for defining water stress (SWC < 10%) according to the dried soil layer classification method [37], the 30-year-old *R. pseudoacacia* in this study did not experience water stress during the two growing seasons (Figure 3). This may be attributed to the relatively adequate water availability during these periods, with 2019 being a wet year and 2020 being a normal water year. After conducting an in-depth analysis of the reasons for the aforementioned differences, we should point out that they are attributable to the distinct study areas and their complex environmental conditions. Research has indicated that planting *R. pseudoacacia* can have a negative impact on local soil moisture when the precipitation is below 600 mm [31]. However, even so, both this study and other scholars’ research have found that 30-year-old *R. pseudoacacia* and *P. orientalis* exhibit strong drought adaptability through their flexible water-use strategies.

This study revealed that, for grasslands, artificial *M. sativa* had higher expenditures of both GW_H_ and GW_I_ than natural *I. cylindrica* grasslands during normal water years. In contrast, during wet years, *I. cylindrica* exhibited greater GW expenditure and storage than *M. sativa*, owing to their lower interception. The study results demonstrate that *I. cylindrica* exhibits more sensitive water use efficiency and stronger drought adaptation capabilities compared to *M. sativa*, which is also supported by the findings of [19]. This advantage is associated with *I. cylindrica* being a perennial herbaceous plant that maintains a well-developed root system, whereas *M. sativa*, due to annual harvesting, does not possess this trait. When comparing long-term restored artificial forests (including *R. pseudoacacia* and *P. orientalis*) with natural grassland dominated by *I. cylindrica*, the latter significantly outperforms in water conservation. Scholars who have conducted research from this perspective have also affirmed water conservation capabilities of adopting entirely natural grassland for restoration [22]. However, based on our research findings and field observations, the vertical stratification height and the reduction in rainfall energy in forestlands are unparalleled by natural grasslands. When rainfall intensity reaches moderate or heavier levels, natural grasslands will be inverted, which decreases surface roughness and increases soil and water loss. Although recovery occurs over time, the water retention capacity is evidently reduced. Therefore, maintaining a proper balance between planted woodlands and natural grasslands is a more reasonable solution.

### 4.2. Effects of Vegetation Type and Regulatory Factors on GW Utilization Efficiency

The expenditure for GW storage includes GW_H_ and GW_L_ [38,39]. The quantities of both are mainly regulated by the demand for evapotranspiration (temperature, wind speed, saturation atmospheric pressure difference, etc.) and the supply of water (GW storage), which, for the demand side, can usually be expressed as a composite indicator of potential evapotranspiration (*ET*_0_) [23]. Since demand and supply often act simultaneously on GW_L_ expenditures, researchers still have controversy about the extent to which each contributes to GW_H_ and GW_L_ [40,41]. However, there is a consensus that evapotranspiration (*ET*) is driven by *ET*_0_ and limited by GW_A_ [13,42]. GW_H_ is essential for vegetation development and, together with GW_L_, represents a key link in the terrestrial water cycle and energy balance, and globally accounts for more than 60% of *ET* in terrestrial ecosystems [43]. In contrast, GW_L_ is considered to have limited ecological improvement, and water managers would like to transform it more into GW_H_, which can be achieved through an appropriate restoration of vegetation [44].

**Figure 6:**
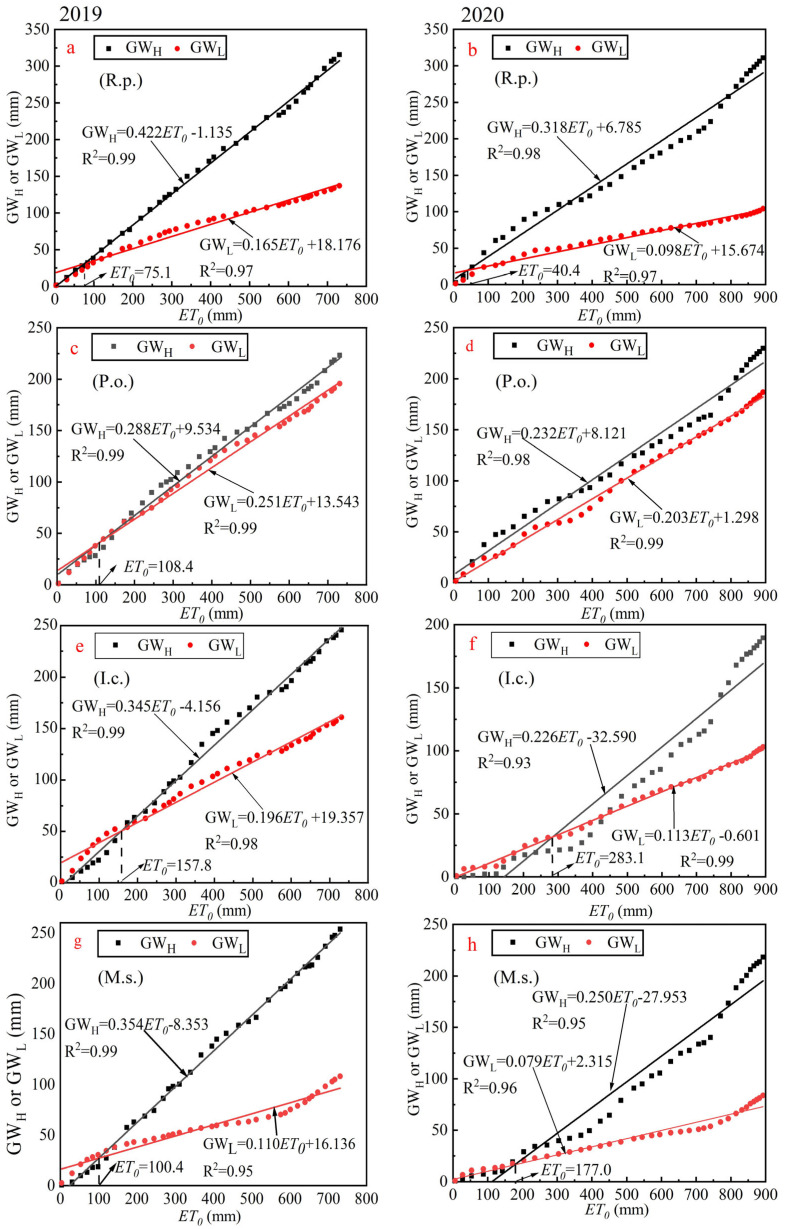
Cumulative plots of GW_H_, GW_L_, and potential evapotranspiration. (**a**,**c**,**e**,**g**) and (**b**,**d**,**f**,**h**) are plots for *R. pseudoacacia*, *P. orientalis*, *I. cylindrica*, and *M. sativa* for the 2019 and 2020 growing seasons, respectively. Cumulative values in the plots were recorded every five days, and the intersection of the two fitted lines in the plots represents the point at which the cumulative values of GW_H_ and GW_L_ are equal in magnitude as *ET*_0_ increases.

Based on the GW_H_ and GW_L_ results for different plots, as shown in Figure 3, there is evident variation in the conversion capacity. Therefore, it is necessary to determine which plant is more efficient in converting under conditions of evaporative demand and water-supply constraints. According to [37], plant growth and development will begin to be limited when soil moisture content is below 10%. Based on the results of our study, it was found that none of the four sample plots experienced water supply limitations during two growing seasons, except for a few days when the average soil moisture content of *P. orientalis* briefly fell below 10%. The GW amount in the four plots is mainly influenced by *ET*_0_ and variations in their conversion capacity. Their relationship is illustrated in Figure 6.

We found that the correlation coefficients of GW_H_ and GW_L_ with *ET*_0_ are greater than 0.9, which indicates they fit very well, and the fit curve is expected to provide a reference for predicting future GW_H_ and GW_L_ transformations. GW_H_ and GW_L_ increased with the increase in *ET*_0_ in both the 2019 and 2020 growing seasons, and the rate of rise of GW_H_ was greater than that of GW_L_. This is also closely related to increased rainfall, which has been demonstrated in arid and semi-arid regions such as Italy and the Hulunbeier Grasslands [45,46]. Based on the slopes of the fitted lines, it can be seen that in both growing seasons, *R. pseudoacacia* had the fastest rate of increase in GW_H_, followed by *M. sativa*. In contrast, *P. orientalis* had the slowest rate of increase in 2019, while *I. cylindrica* showed the slowest rate in 2020. Both sides of the intersection of the fitted lines indicate that GW_H_ and GW_L_ are differentially dominant when *ET* demand is low. Then, the intersection point is the threshold for the transition of dominance between GW_H_ and GW_L_. Across the two growing seasons, the conversion thresholds for GW_H_ and GW_L_ dominance for *R. pseudoacacia* were 75.1 and 40.4 mm, respectively. For *P. orientalis*, the threshold was 108.4 mm in 2019 (note that in 2020, *P. orientalis* consistently had more GW_H_ than GW_L_, which may be related to the fact that *P. orientalis* is an evergreen plant, so no clear threshold was observed). For *I. cylindrica*, the thresholds were 157.8 and 283.1 mm; for *M. sativa*, they were 100.4 and 177.0 mm, respectively.

From the thresholds (Figure 6), it is easy to find that for woodland in 2020, when plant growth and development were limited not by moisture but by the drier and hotter conditions (with precipitation being less in the 2020 growing season than in 2019 and *ET*_0_ greater than in 2019), the time at which GW_H_ dominated GW expenditure was significantly earlier. Woodland may be able to use as much of the limited water as possible by regulating its own growth and development to minimize inefficient consumption [47]. This adjustment ability is clearly ahead of other vegetation. Grasslands opted for a different strategy. Drier and hotter conditions in the early growth stage slow down the development of *I. cylindrica* and *M. sativa*, which results in more GW_A_ being dissipated as GW_L_. This condition continued until the mid-growth stage. Comparing the two grasses, *I. cylindrica* has more room for improvement in its ability to convert GW_H_, but that may be the secret to its ability to be a natural grass species.

In summary, woodland demonstrated significantly superior efficiency in GW utilization compared to grassland, further corroborating its greater resilience to climate change, with *R. pseudoacacia* showing the best overall performance. However, *P. orientalis* is likely to be more suitable when moisture is limited due to its lower GW expenditures and stable, efficient GW use. *I. cylindrica* and *M. sativa*, as typical representatives of natural and artificial grassland, respectively, can only passively adapt to the natural environment. The proportion of GW_L_ will likely continue to expand as moisture is further reduced, but the total GW expenditure is less than for woodland.

### 4.3. Recommendations for Optimizing Vegetation for GW Management

**Figure 7:**
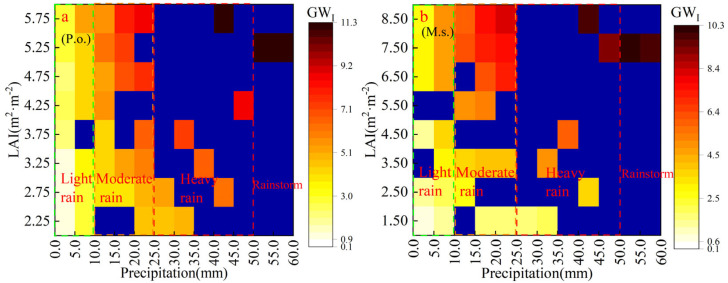
Thermogram of GW_I_ versus rainfall and LAI ((**a**) is *P. orientalis* sample plot, (**b**) is *M. sativa* sample plot).

The results of GWCs (Figure 4) show that during precipitation recharge to GW, the interception of vegetation canopy resulted in the production of more GW_I_, which reduces the recharge of GW_A_. *P. orientalis* had the most GW_I_ in the woodland, with 198.4 and 175.5 mm in the 2019 and 2020 growing seasons, totaling 69.2% more than *R. pseudoacacia* for the same rainfall. *M. sativa* had the most GW_I_ in the grassland, with 177.9 and 161.1 mm, totaling 5.3% more than *I. cylindrica* (Figure 7). The production of GW_I_ reduces the actual amount of rain reaching the ground and the effective replenishment of GW_A_, resulting in an unproductive loss of GW resources [48,49]. However, GW_I_ retained by vegetation also weakens rainfall’s impact on the ground and prevents soil erosion [50,51]. So, how can we maximize the reduction in GW_I_ without weakening its function in preventing soil erosion?

In the rain-fed agriculture area of the LP, precipitation is the most important factor affecting interception, followed by LAI; the rest have little effect [52]. Therefore, a reasonable solution to reduce GW_I_ is expected to be found by exploring how rainfall and LAI affect GW_I_. Based on the measured LAI of *P. orientalis* and *M. sativa* samples in the growing season and the results of field rainfall interception, the relationship established is shown in Figure 7.

By observing Figure 6a,b, it can be inferred that the amount of GW_I_ of *P. orientalis* and *M. sativa* is directly proportional to rainfall and LAI. The interception generally shows a linear increasing trend from the lower left to the upper right corner. However, it should be noted that when the rainfall is greater than 10 mm, the effect of LAI on the amount of GW_I_ changes under different rainfall conditions. As rainfall continues to increase, the effect of LAI on interception also seems to increase. Under the same rainfall situation, although the LAI of *M. sativa* is greater than that of *P. orientalis*, the GW_I_ is lower in *M. sativa* than in *P. orientalis*. This is due to the fact that *P. orientalis*, being an evergreen coniferous forest species with greater canopy thickness than *M. sativa* and *R. pseudoacacia*, offers greater resistance to rainfall penetration and is thus able to intercept more rainfall.

To investigate whether the effect of LAI on GW_I_ consistently grows across various rainfall classes, this study categorized the daily rainfall into four groups: light rain, moderate rain, heavy rain, and rainstorm (as depicted in Figure 7). Multiple linear regression analyses were performed, and the findings are presented in Table 4.

It can be seen from the t-values of each of *P* and LAI and the coefficients of the regression equations that the degree of *P* influence on GW_I_ was greater than that of LAI, which is the same as the results of the study by [22] findings. Categorizing the rainfall, it was found that *P* and LAI had a significant effect on GW_I_ (*p* > 0.01). The effect of *P* on GW_I_ was greatest when the rainfall level was “light rain”. When *P* was above moderate rainfall, the t-value showed that the effect of *P* on GW_I_ was decreasing. Meanwhile, the effect of LAI continued to increase, especially in the case of heavy rainfall and rainstorms, where the t-value was more than three times that of light rainfall, and LAI had the greatest effect on GW_I_. The results of the regression coefficients show that every 12% increase in LAI can increase GW_I_ by 1 mm, while rainfall only needs to increase by 8% to achieve the same effect. Obviously, the effect of *P* on the amount of GW_I_ remains the greatest for the *M. sativa* sample plot. Similarly, from the t-values, it can be observed that the effect of LAI on GW_I_ continued to increase with the increase in rainfall level. When the rainfall level reached heavy rainfall, LAI also became the most influential factor of GW_I_.

Combining the results of the above analyses with rainfall and vegetation monitoring data, we find that in the early growth stage, when the vegetation is less developed and rainfall is low, there is less GW_I_ to intervene. In the middle and late growth stages, the total rainfall and LAI increased significantly, and the amount of GW_I_ increased, which is also a frequent period of soil erosion and should be the main period to reduce GW_I_ [48]. Rainfall is the most important factor causing soil erosion, but not all rainfall produces erosion, and the rainfall threshold for producing soil erosion varies in different areas [53]. In the NXHG Basin, the rainfall threshold for erosion is 21.0 mm for woodlands and 23.7 mm for *M. sativa* [53]. The monitoring data showed that the rainfall type of the 2019–2020 growing season in the NXHG Basin was mainly light and moderate rainfall, which accounted for 88.2% of the total number of rainfall events, and the maximum of moderate rainfall was 24.9 mm/24 h. This rainfall value should then be the boundary condition for balancing the reduction in GW_I_ and the prevention of erosion.

Then, considering the rainfall threshold for soil erosion, the LAI should be reduced to ensure that *P. orientalis* and *M. sativa* maintain a minimum interception capacity of 4 mm and 1.3 mm, respectively, at a rainfall of 24.9 mm. Since GW_I_ with *P* and LAI are positively correlated, we can assume that LAI at this level is the optimal value for *P. orientalis* and *M. sativa* to achieve the minimum GW_I_ and soil conservation balance under light and moderate rainfall. It should be noted that reducing the LAI by manual pruning requires that the pruned branches and leaves be covered to the ground [54,55], so the rainfall threshold for sediment production of the sample plots will not be reduced to ensure the reliability of the study.

To determine the appropriate value for which the LAI can be reduced, the above rainfall and different LAI were input into the model for trial calculations. The initial conditions were set to the soil moisture content on June 1, and the rest of the settings were the same as in the previous section. The model’s output shows that the LAI of *P. orientalis* and *M. sativa* should be trimmed to 2.1 and 1.0 m^2^·m^−2^, respectively.

## 5. Conclusions

We conducted field experiments and simulations to study the GW heterogeneity of four typical long-term restored vegetation types on the Loess Plateau. The main conclusions are as follows:

For forestland, compared to *R. pseudoacacia*, *P. orientalis* had lower values of GW_H_ and GW_A_ in both growing seasons, while GW_L_ and GW_I_ were higher. For grassland, compared with *M. sativa*, overall, *I. cylindrica* had more GW_L_ but less GW_H_, GW_A_, and GW_I_. Combined with the GWCs data, it can be seen that *P. orientalis* and *M. sativa* should be preferred for local vegetation restoration in terms of reducing GW_A_ consumption on rain-free days, and *R. pseudoacacia* and *I. cylindrica* are more recommended in terms of their water conservation function on rainy days. Across the two growing seasons, the conversion thresholds of *ET*_0_ for GW_L_ to GW_H_ dominance for *R. pseudoacacia* were 75.1 and 40.4 mm, respectively. For *P. orientalis*, the threshold was 108.4 mm in 2019 (note that in 2020, *P. orientalis* consistently had more GW_H_ than GW_L_, which may be related to the fact that *P. orientalis* is an evergreen plant, so no clear threshold was observed). For *I. cylindrica*, the thresholds were 157.8 and 283.1 mm; for *M. sativa*, they were 100.4 and 177.0 mm, respectively.

We are convinced that detailed field observations, combined with GW numerical modeling, can identify the consequences of changes in climatic parameters and vegetation species, which can be used to devise appropriate soil and water conservation strategies and improve water management in water-limited areas.

## Figures and Tables

**Table 1:** Vegetation parameters of trees.

Species	Height (cm)	DBH (cm)	Canopy Amplitude (cm)	Coverage (%)	Density	Altitude (m)	Slope (°)	Topography
*R* *. pseudoacacia*	620.0 ± 45.0	16.1 ± 1.3	400.4 ± 75.5	71.5	3.0 m × 3.0 m	1211.6	25.0	sloping terrace
*P. orientalis*	534.0 ± 32.5	17.5 ± 1.8	240.5 ± 42.8	92.2	3.0 m × 3.0 m	1235.7	26.1	sloping terrace

**Table 2:** Vegetation parameters of grass.

Species	Height (cm)	Coverage (%)	Altitude (m)	Slope (°)	Topography
*I. cylindrica*	46.4 ± 8.8	89.8	1211.6	31.0	sloping terrace
*M. sativa*	50.2 ± 6.5	88.6	1222.4	33.2	sloping terrace

**Table 3:** Sensitivity ranking and calibrated results of model parameters for each plot.

Parameter	Description	Range of Value	Order of Sensitivity				Calibrated Results			
			*R. P.*	*P. O.*	*I. C.*	*M. S.*	*R. P.*	*P. O.*	*I. C.*	*M. S.*
*θ_r_*_2_/(cm^3^·cm^−3^)	Residual moisture content (40∼100 cm)	0.01~0.05	11	10	11	12	0.0420	0.0630	0.0565	0.0538
*θ_s_*_1_(cm^3^·cm^−3^)	Saturated moisture content (0∼40 cm)	0.25~0.80	3	4	3	3	0.4818	0.4612	0.4928	0.4778
*θ_s_*_2_/(cm^3^·cm^−3^)	Saturated moisture content (40∼100 cm)	0.25~0.80	5	7	6	7	0.5069	0.4957	0.4872	0.4972
*α*_1_/cm^−1^	Reciprocal of inlet air suction (0∼40 cm)	0.002~0.1	10	11	10	10	0.0246	0.0144	0.0167	0.0208
*α*_2_/cm^−1^	Reciprocal of inlet air suction (40∼100 cm)	0.002~0.1	13	13	13	13	0.0376	0.0138	0.0174	0.0228
*n*_1_	Parameter of pore size distribution (0∼40 cm)	1.1~2	2	3	1	1	1.2022	1.1945	1.1620	1.2258
*n*_2_	Parameter of pore size distribution (40∼100 cm)	1.1~2	1	1	2	2	1.1965	1.2272	1.1448	1.2459
*K_s_*_1_/(cm·d^−1^)	Saturated hydraulic conductivity (0∼40 cm)	0~750	12	12	12	11	55.4	58.1	43.2	88.9
*μ*	Extinction coefficient	0~1	7	2	4	4	0.46	0.33	0.36	0.49
*a*	The empirical parameter of the interception module	0~1	4	5	5	8	0.09	0.28	0.16	0.16
*h*_1_/cm	Upper optimum potential threshold of root water uptake	−2000~0	6	9	8	5	−265.7	−392.6	−442.5	−658.8
*h*_2_/cm	Lower optimum potential threshold of root water uptake	−10,000~0	8	8	9	6	−885.4	−1205.2	−1202.4	−1992.6
*h*_3_/cm	Soil water potential for permanent wilting	−60,000~0	9	6	7	9	−26,134.8	−20,285.4	−9125.6	−1148.6

Note: *θ_r_*, *θ_s_*, *α*, *n*, and *K_s_* are basic parameters in van Genuchten–Mualem model; *μ* and *a* are parameters in beer equation and interception module, respectively. *h*_1_, *h*_2_, and *h*_3_ are characteristic water potential in the water stress response function; *R. P.*, *P. O.*, *I. C.*, and *M. S.* are *R. pseudoacacia*, *P. orientalis*, *I. cylindrica*, and *M. sativa*, respectively.

**Table 4:** Multiple linear regression analysis results of GW_I_, rainfall, and LAI.

Plot	Precipitation Level	Regression Equation	R^2^	t		
				*P*	*LAI*	Constant
*P. orientalis*	Light rain	GW_I_ = 0.380 *P* + 0.484 *LAI −* 1.216	0.98	30.635 **	16.223 **	−10.397 **
	Moderate rain	GW_I_ = 0.170 *P* + 1.096 *LAI −* 1.599	0.96	14.620 **	23.240 **	−5.409 **
	Heavy rain and rainstorm	GW_I_ = 0.057 *P* + 1.724 *LAI −* 1.145	0.99	15.129 **	57.924 **	−10.676 **
	Whole	GW_I_ = 0.164 *P* + 0.940 *LAI −* 1.629	0.89	21.317 **	11.704 **	−4.661 **
*M. sativa*	Light rain	GW_I_ = 0.325 *P* + 0.339 *LAI −* 0.794	0.92	12.273 **	13.171 **	−4.607 **
	Moderate rain	GW_I_ = 0.121 *P* + 0.777 *LAI −* 1.072	0.95	6.094 **	21.849 **	−2.610 *
	Heavy rain and rainstorm	GW_I_ = 0.034 *P* + 1.137 *LAI* − 0.635	0.99	4.418 **	38.917 **	−2.640 *
	Whole	GW_I_ = 0.125 *P* + 0.592 *LAI* − 0.860	0.84	13.681 **	14.491 **	−3.180 **

Note: “**” indicates passing *F*_0.01_ significance test, and “*” indicates passing *F*_0.05_ significance test.

## Data Availability

Data are contained within the article.
